# Horizontal transmission and recombination of *Wolbachia* in the butterfly tribe Aeromachini Tutt, 1906 (Lepidoptera: Hesperiidae)

**DOI:** 10.1093/g3journal/jkab221

**Published:** 2021-07-01

**Authors:** Zimiao Zhao, Jianqing Zhu, Ary A Hoffmann, Lijun Cao, Li Shen, Jie Fang, Shuojia Ma, Qunxiu Liu, Weidong Yu, Liying Tang, Yongqiang Wang, Weibin Jiang

**Affiliations:** 1 Laboratory of Environmental Entomology, College of Life Sciences, Shanghai Normal University, Shanghai 200234, People’s Republic of China; 2 Shanghai Zoological Park, Shanghai 200335, People’s Republic of China; 3 School of BioSciences, Bio21 Institute, The University of Melbourne, Parkville, VIC 3052, Australia; 4 Institute of Plant and Environmental Protection, Beijing Academy of Agriculture and Forestry Sciences, Beijing 100097, People’s Republic of China

**Keywords:** Aeromachini, *Wolbachia*, divergence time, cophylogeny, recombination, horizontal transmission

## Abstract

*Wolbachia* is arguably one of the most ubiquitous heritable symbionts among insects and understanding its transmission dynamics is crucial for understanding why it is so common. While previous research has studied the transmission pathways of *Wolbachia* in several insect lineages including Lepidoptera, this study takes advantage of data collected from the lepidopteran tribe Aeromachini in an effort to assess patterns of transmission. Twenty-one of the 46 species of Aeromachini species were infected with *Wolbachia*. Overall, 25% (31/125) of Aeromachini specimens tested were *Wolbachia* positive. All *Wolbachia* strains were species-specific except for the *w*Jho strain which appeared to be shared by three host species with a sympatric distribution based on a cophylogenetic comparison between *Wolbachia* and the Aeromachini species. Two tests of phylogenetic congruence did not find any evidence for cospeciation between *Wolbachia* strains and their butterfly hosts. The cophylogenetic comparison, divergence time estimation, and *Wolbachia* recombination analysis revealed that *Wolbachia* acquisition in Aeromachini appears to have mainly occurred mainly through horizontal transmission rather than codivergence.

## Introduction

*Wolbachia* is the most widespread endosymbiotic bacterium that infects a large variety of arthropods and filarial nematodes (Bandi *et al.* 1998; Weinert *et al.* 2015). In butterflies, *Wolbachia* infections have been reported in five families (Papilionidae, Hesperiidae, Nymphalidae, Pieridae, and Lycaenidae) so far (Jiggins *et al.* 2000; Dyson *et al.* 2002; Hiroki *et al.* 2004; Tagami and Miura 2004; Russell *et al.* 2009; Bipinchandra *et al.* 2012; Jiang *et al.* 2018) . The transmission pattern of *Wolbachia* is predominantly vertical and secondarily horizontal (Raychoudhury *et al.* 2009). It induces various reproductive alterations to alter host biology, like cytoplasmic incompatibility (CI), male killing (MK), feminization induction (FI), and thelytokous parthenogenesis (Yen and Barr 1971; Rousset *et al.* 1992; Stouthamer *et al.* 1993; Hurst and Jiggins 2000). In butterflies, some of these effects are well established, especially MK in *Hypolimnas bolina* and *Acraea encedon* (Jiggins *et al.* 2001; Dyson and Hurst 2004), CI in *H.* *bolina* and *Polygonia calbum* (Hornett *et al.* 2008; Kodandaramaiah *et al.* 2011) and FI in *Eurema hecabe* (Kageyama *et al.* 2008).

Based on phylogenetic reconstructions with a set of loci (MLST) used to type *Wolbachia* strains, *Wolbachia* fall into 17 supergroups designated by the letters A–R, with supergroup G being controversial (Baldo and Werren 2007; Augustinos *et al.* 2011; Wang *et al.* 2016). *Wolbachia* in butterflies has been associated only with supergroups A and B. *Wolbachia* from supergroup B occurs in a wide range of butterfly hosts and an MLST allele (ST-41) is core in butterfly hosts worldwide (Bipinchandra *et al.* 2012; Ilinsky and Kosterin 2017). While *Wolbachia* has been investigated in detail in some infected butterfly species (Hornett *et al.* 2006; Charlat *et al.* 2007; Narita *et al.* 2007; Gompert *et al.* 2008; Duplouy *et al.* 2010; Jiang *et al.*^2014^, ^2016^), there are few systematic studies of *Wolbachia* at the molecular level across a group of related species even though such an analysis can be useful in assessing horizontal transmission patterns in other insects such as *Drosophila* (Turelli *et al.* 2018), *Agelenopsis* (Baldo *et al.* 2008), *Trichogramma* (Huigens *et al.* 2004), *Rhagoletis* (Schuler *et al.* 2013), and *Altica* (Jackel *et al.* 2013). In this study, we tackle this issue by evaluating the molecular phylogeny of the tribe Aeromachini and associating it with phylogenetic patterns for *Wolbachia* infections to assess patterns of transmission.

Aeromachini is a tribe of family Hesperiidae and currently comprises 136 described species in 11 genera (Warren *et al.* 2008; 2009; Huang 2009; Li *et al.* 2019). Most species are restricted geographically to the Oriental Region, and a few species are found in Afrotropical Region and Palearctic Region (Eliot 1969). The common ancestor of this tribe was inferred to originate in Southeast Asia (Huang *et al.* 2019). We have reported the external features and the molecular phylogeny of the tribe in a preliminary study (Li *et al.* 2019). In prior screening, we found *Wolbachia* in *Aeromachus inachus*, *A.* *virgata*, and *Halpe dizangpusa* which prompted our further study of all Aeromachini species in China.

In this study, we characterized the *Wolbachia* in tribe Aeromachini by MLST genotyping. Furthermore, we conducted a cophylogenetic analysis between *Wolbachia* and their Aeromachini hosts, compared the age of *Wolbachia* divergence with that of host species, and analyzed the actual and potential recombination of *Wolbachia* in Aeromachini to provide information on the patterns of *Wolbachia* transmission across this tribe.

## Materials and methods

### Samples collection, DNA extraction, and *Wolbachia* MLST typing

**Figure 1:**
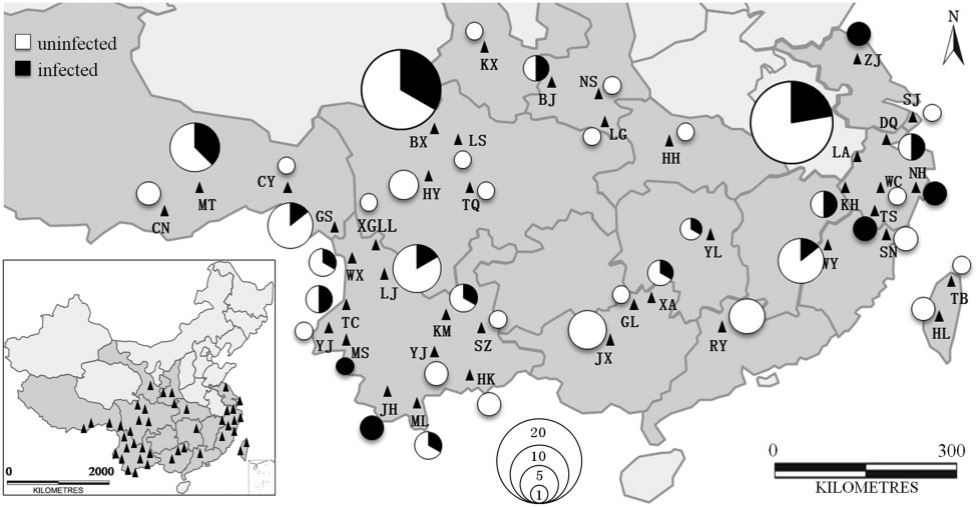
The distribution of specimens collected in China infected or uninfected with *Wolbachia*. The sizes of the circles are directly proportional to the number of individuals analyzed (black: infected with *Wolbachia*, white: uninfected). The triangles refer to the location of collection sites and the letters are the Abbreviation of place names. For full site names and other details, please see Supplementary Table S1.

We collected a total of 125 Aeromachini butterflies representing 10 genera and 46 species from 42 local regions in China across the last 12 years (Figure 1 and Supplementary Table S1). All specimens were caught with sweep nets and saved in small envelopes. The species were identified with morphological characteristics and molecular techniques (Jiang *et al.* 2019; Li *et al.* 2019). The DNA was isolated from whole abdomens of specimens using a QIAamp DNA Mini kit (Qiagen, Hilden, Germany).

To screen for *Wolbachia* infection status, the *wsp* locus was amplified followed the published protocols described by Zhou *et al.* (1998; Supplementary Table S2). The characterization of *Wolbachia* strains was performed to sequence multiple loci suggested by *Wolbachia* MLST database (http://pubmlst.org/wolbachia) (Zhou *et al.* 1998; Supplementary Table S2). The MLST typing consisted of five *Wolbachia* gene fragments (*gatB*, *coxA*, *hcpA*, *ftsZ*, and *fbpA*). The PCR product was purified using the Wizard SV Gel and PCR Clean-up System (Promega, Madison, WI, USA). The purified product was ligated with the pGEM-T easy vector (Promega, Madison, WI, USA) using a ligation mix (TaKaRa). Competent cells (*Escherichia coli* JM109, TaKaRa) were then transformed with the plasmid. Plasmid DNA was extracted using the Pure Yield Plasmid Miniprep System (Promega, Madison, WI, USA). The sequencing was performed using an ABI 377 automated DNA sequencer.

A Mantel test was used to compare *Wolbachia* frequency (pooled across species) and geographical distribution of their corresponding Aeromachini hosts with the software Isolation by Distance (IBD; Bohonak 2002). It was performed on the pairwise node distance matrix of *Wolbachia* frequency and host Aeromachini species to test for an association between matrices (Maddison 2015).

### Cophylogenetic analysis

The MLST sequences were aligned with outgroups retrieved from the MLST database (host: *Brugia malayi*, *Cordylochernes* *scorpioides*, and *Opistophthalmus capensis*; Supplementary Table S1) using Bioedit v. 7.0 (Hall 1999). The HKY + I model was selected as the best-fit substitution model with PartitionFinder v2.1.1 (Lanfear *et al.* 2012) using the Bayesian Information Criterion (BIC). Maximum likelihood (ML) tree was constructed with the concatenated data using IQtree 1.4.2 (Nguyen *et al.* 2015). To assess nodal support, we performed 1000 ultrafast bootstrap replicates with UFBoot and an SH-aLRT test with 1000 replicates (Hoang *et al.* 2018).

For the molecular phylogenetic constructions of Aeromachini species (the concatenated mitochondrial and nuclear genes), we retrieved the mitochondrial genes COI, COII, and three variable domains of the nuclear DNA (D3 region of 28S rDNA, V4 and V7 regions of 18S rDNA) from GenBank (MK344780–MK345418). The method of ML tree construction follows that used for the hosts as described above. The GTR + G model was selected as the best-fit substitution model for this dataset.

A Mantel test was used to compare genetic and *Wolbachia* distance matrices with IBD (Bohonak 2002). It was performed on the pairwise node distance matrix of *Wolbachia* strains and host Aeromachini species to test for an association between matrices (Hall 1999). Another test of phylogenetic congruence between butterflies and endosymbiont partners was undertaken with the Procrustean Approach to Cophylogeny (PACo; Balbuena *et al.* 2013). The analysis was performed in R with 100,000 permutations using packages VEGAN v.2.4.6 (Oksanen *et al.* 2018) and APE v.4.1 (Paradis *et al.* 2004).

### Estimation of divergence time

We referred to a molecular dating analysis of *Wolbachia* supergroups A and B to compare the divergence times of *Wolbachia* (Gerth and Bleidorn 2016) with the age of Aeromachini species divergence. The divergence times of all *Wolbachia*-infected Aeromachini species were inferred with the relaxed-clock molecular dating estimation by BEAST 1.5.2 (Excoffier *et al.* 2005). The HKY model of nucleotide substitution with gamma distributed rate variation among sites was used to analyze and the Yule speciation method was assumed. We used the age ranges estimated from Chazot *et al.* (2019) to calibrate the split between Hesperiidae and Hedylidae (81–114 Mya) and the age ranges between Hesperiinae and Heteropterinae (35–55 Mya). We also used a recently described fossil hesperiid, *Pamphilites abdita* Scudder, 1875 to constrain the minimum stem age of subfamily Hesperiinae to 25 Mya (de Jong 2016). Chains were run for 50 million generations, with the first 20% discarded as burn-in. The results were summarized with TRACER 1.5 (Fu and Li 1993).

### Recombination analysis

Gene recombination can interfere with and mislead phylogenetic relationships of species. We detected recombination events with the MLST and *wsp* genes, to clarify whether horizontal transmission had occurred among these *Wolbachia* strains. To examine recombination among *Wolbachia* strains from Aeromachini species, each MLST gene and *wsp* gene were detected using RDP3 (Martin *et al.* 2010). Seven methods (RDP, GENECONV, BootScan, MaxChi, Chimaera, SiScan, and 3Seq) in program RDP3 were chose to identify the recombinant sequences and recombination breakpoints. The potential recombination events can be detected by any of the methods listed above. As recommended for this procedure, the breakpoint positions and recombinant sequences inferred from every potential recombination event were manually checked and adjusted following the phylogenetic and recombination signal analysis features available in RDP3.

To visualize potential recombination events, ML trees for each MLST gene and *wsp* were constructed with 10 reference STs and 3 outgroups retrieved from the MLST database (Supplementary Table S1) using IQtree 1.4.2 (Nguyen *et al.* 2015). They were checked for their supergroup clustering in ML trees. A potential recombination event could be found from inconsistencies between gene trees (Werren and Bartos 2001; Baldo *et al.* 2006).

## Results

### Infection rates and diversity of *Wolbachia*

In the examined butterflies, 25% (31/125) of samples were *Wolbachia* positive and 46% (21/46) of Aeromachini species in this study were considered infected with *Wolbachia*, with some of these shown to be polymorphic for the infection despite limited sampling. The infection status and geographical distribution of each sample and species is shown in Figure 1, Supplementary Tables S1 and S3. The Mantel test analysis indicated a nonsignificant correlation between *Wolbachia* frequency and geographic location of their corresponding Aeromachini hosts when pooled across species and samples (*r* = 0.1714, *P* *=* 0.060), suggesting a weak spatial structure in the incidence of *Wolbachia*. However, there is no obvious association between *Wolbachia* frequency overall and latitude (Figure 1), a pattern previously noted for moths (Ahmed *et al.* 2015). We amplified five MLST loci to characterize *Wolbachia* strains. Each of the five MLST genes and the *wsp* gene detected from each Aeromachini species had the same sequence. The strains are denoted based on the MLST loci as *w*Pic, *w*Mag, *w*Ina, *w*Kyn, *w*Jho, *w*Yin, *w*Lua, *w*Dio, *w*Hyr, *w*Bai, *w*Lin, *w*Vir, *w*Pes, *w*Dol, *w*Lat, *w*Sub, *w*Kua, *w*Diz, and *w*Str (GenBank accession numbers: MT935975–MT936085).

### Comparison of *Wolbachia* and Lepidoptera phylogenies

**Figure 2:**
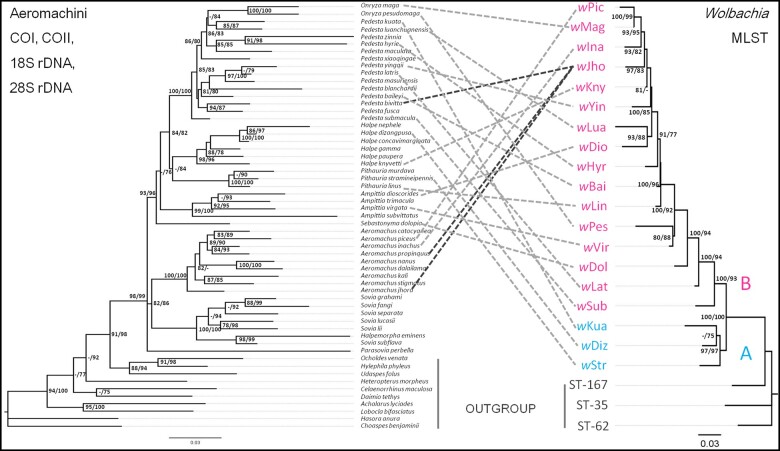
Cophylogenetic analysis of Aeromachini based on mtDNA + nDNA (left) and corresponding *Wolbachia* strains based on MLST (right). Numbers beside nodes are IQTREE ultrafast bootstrap and SH-aLRT values. The *Wolbachia* strains of Supergroups A are in blue and those of Supergroups B are in red. Scale bars indicate the mean number of substitutions per site.

All *Wolbachia* strains were species specific except for *w*Jho shared by three host species (*Aeromachus jhora*, *Aeromachus propinquus*, and *Pedesta bivitta*) sympatric in Yunnan Province, southwest China (Figure 2). Although the concatenated sequences of hosts and *Wolbachia* strain types matched well, the topologies of Aeromachini hosts and corresponding *Wolbachia* strains (which fell into supergroups A and B) were not congruent (Figure 2). It is possible that coevolution could have occurred between hosts and their *Wolbachia* in the Aeromachus clade, although the Mantel test indicated no significant correlation between the genetic distances of the *Wolbachia* strains and their host Aeromachini species (*r* = −0.094, *P* = 0.719). This points to the horizontal transmission being an important mode of transmission. Similarly, PACo provided no evidence for congruence between the phylogeny of Aeromachini and that of their endosymbionts (PACo m^2^ = 0.033, *P* *=* 0.402).

### Divergence time estimation

**Figure 3:**
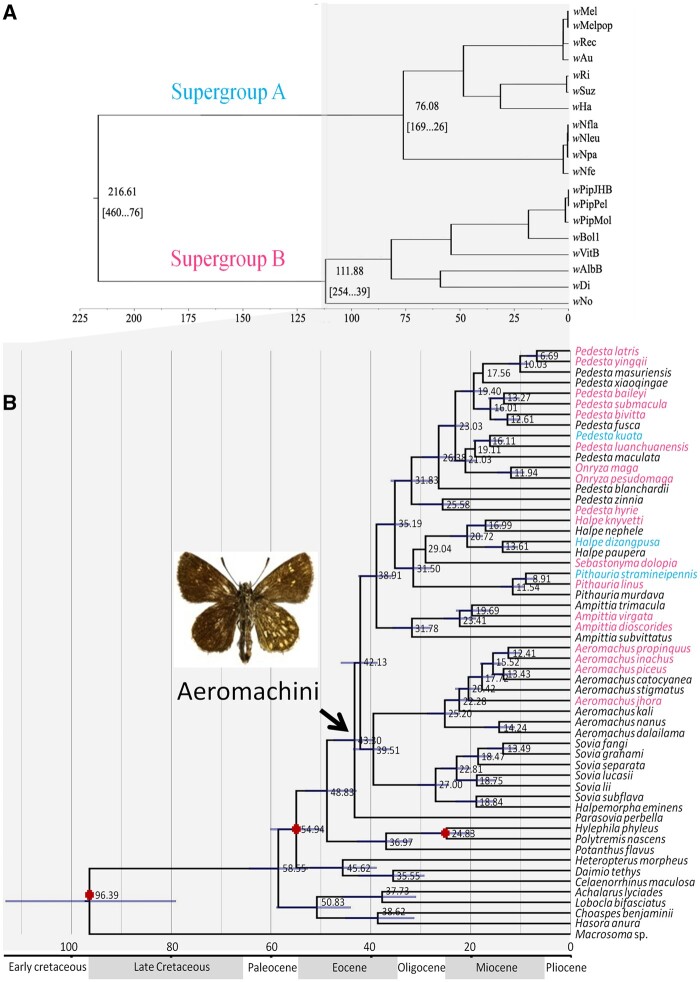
(A) Estimated divergence times of *Wolbachia* Supergroups A and B based on Gerth and Bleidorn (2016), and (B) Bayesian Inference (BI) tree of mtDNA datasets for Aeromachini species using uncorrelated lognormal relaxed clock in BEAST v1.5.2. Posterior probabilities of nodes are shown to the right of the node branch when higher than 0.95. The violet bars (B) indicate 95% highest posterior density interval (HPD) of the node ages.

Divergence time of the Aeromachini was estimated with the relaxed clock molecular dating implemented in BEAST. We compared the divergence between *Wolbachia* supergroups based on genomic data (Gerth and Bleidorn 2016) with divergence times of Aeromachini and found the youngest divergence between species at 6.69 Mya (8.82–4.03, 95% HPD) and the oldest gap between *Parasovia perbella* and the other species at 43.30 Mya (47.93–39.61, 95% HPD) (Figure 3).

### Recombination of MLST and *wsp* genes

The recombination analysis within each MLST gene and *wsp* gene showed that the polymorphic sites of the alignment of the *FtsZ* alleles are not randomly distributed, but a mosaic pattern consistent with recombination in a coinfected host. To estimate the approximate recombination events, all events were confirmed with five of seven RDP3 algorithms (Table 1). The *FtsZ* sequence of four *Wolbachia* strains (*w*Ina from *A.* *inachus*; *w*Jho from *A.* *jhora*, *A.* *propinquus*, and *P.* *bivitta*; *w*Yin from *Pedesta yingqii*; and *w*Dol from *Sebastonyma dolopia*) are the same recombinant between *Wolbachia* strain *w*Lat detected from *Pedesta latris* and *Wolbachia* strain *w*Dio from *Ampittia dioscorides* (Supplementary Figure S1).

**Table 1:** Average P-values of recombinations estimated using the RDP3 program

Recombination strains	Average P-value						
	RDP	GENECONV	BootScan	MaxChi	Chimaera	SiScan	3Seq
*w*Ina	5.306 × 10^−09^	2.475 × 10^−08^	5.032 × 10^−10^	8.266 × 10^−11^	7.207 × 10^−12^	—	1.395 × 10^−18^
*w*Jho	—	1.585 × 10^−06^	1.516 × 10^−08^	5.784 × 10^−11^	5.719 × 10^−11^	—	8.139 × 10^−18^
*w*Yin	—	1.308 × 10^−10^	1.904 × 10^−12^	2.522 × 10^−11^	7.657 × 10^−12^	—	1.177 × 10^−18^
*w*Dol	—	1.585 × 10^−16^	2.550 × 10^−09^	5.784 × 10^−11^	5.784 × 10^−11^	—	5.360 × 10^−18^

**Figure 4:**
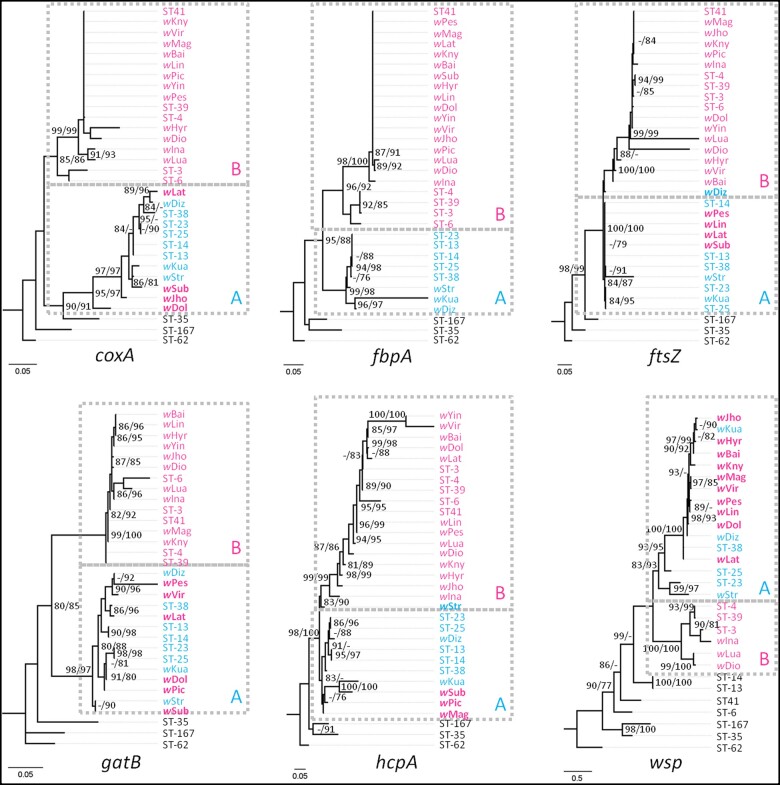
Maximum likelihood trees for each MLST gene and the *wsp* gene. Numbers beside nodes are IQTREE ultrafast bootstrap and SH-aLRT values. The *Wolbachia* strains of Supergroups A are in blue and those of Supergroups B are in red.

We also reconstructed ML trees for each MLST gene and the *wsp* gene separately (Figure 4). Eleven of the nineteen *Wolbachia* strains (*w*Jho, *w*Pic, *w*Mag, *w*Lin, *w*Vir, *w*Pes, *w*Dol, *w*Lat, *w*Sub, *w*Diz, and *w*Str) were found to have inconsistent supergroup allocation among the five MLST gene trees. For example, the localization of *w*Jho on the ML tree was with the B-supergroup (Figure 2). This was associated with a *coxA* allele that belonged to supergroup A, in contrast to alleles at other loci belonging to supergroup B (Figure 4). Therefore, there was substantial incongruence between the *Wolbachia* phylogenies based on the MLST genes and the *wsp* gene sequences (Figure 4) and highlights limitations of supergroup assignment.

## Discussion

Two reports have predicted the incidence of *Wolbachia* in lepidopteran insects and arthropods more generally (Weinert *et al.* 2015; Ahmed *et al.* 2016). The estimated infection incidence in species was predicted to be 80% in Lepidoptera, which is much higher than the 52% incidence predicted in arthropods. However, the mean prevalence of *Wolbachia* in Lepidoptera (27%) is similar to that that in arthropods (24%). The high incidence and low prevalence of *Wolbachia* in Lepidoptera was interpreted as indicating substantial horizontal transmission of *Wolbachia* (Ahmed *et al.* 2016). For the Aeromachini butterflies considered in this study, the mean prevalence in samples (25%) was like the value in other Lepidoptera (27%) and arthropods more generally (24%). On the other hand, the presence of the infection at the species level (46%) was similar to that in arthropods (52%) but considerably lower than reported previously in Lepidoptera (80%). However, the 21 uninfected species in this study are often represented by only 1 or 2 individuals, such as *Ampittia trimacula*, *A.* *jhora*, *Pedesta xiaoqingae*, and *Pedesta zinnia*. The proportion of species infected should therefore be considered as an underestimate of the actual incidence of *Wolbachia* infection across Aeromachini species until larger sample sizes across the geographic range of species are considered.

Two cophylogenetic analyses revealed no correlation of genetic distances between *Wolbachia* strains and their butterfly hosts, which further supports horizontal transmission of *Wolbachia* in the tribe. The divergence time of *Wolbachia* supergroups was compared with that of Aeromachini species (Figure 3). Gerth and Bleidorn (2016) estimated the divergence time between *Wolbachia* supergroups A and B was 216.61 Mya. This implies that transfers of *Wolbachia* from different supergroups between Aeromachini species cannot due to divergence coinciding with speciation events which are dated between 6.69 and 43.30 Mya. Instead, these analyses point to clear cases of horizontal transmission. The *Wolbachia* strain *w*Jho provides a particularly strong argument for horizontal transmission, given that it was present in three species in the tribe (Figure 2). The individuals of *A.* *jhora*, *A.* *propinquus*, and *P.* *bivitta*, infected with *w*Jho, co-occur in Yunnan Province, southwest of China, presumably reflecting an opportunity for horizontal transmission.

Pathways of horizontal transmission for *Wolbachia* could occur through hybridization (*e.g.*, Jiang *et al.* 2018), feeding on common plants (*e.g.*, Sintupachee *et al.* 2006; Li *et al.* 2017), ectoparasitic mites (*e.g.*, Jaenike *et al.* 2007; Gehrer and Vorburger 2012), or parasitoids (*e.g.*, Vavre *et al.* 1999; Ahmed *et al.* 2015). To our knowledge, there is no report of hybridization in the tribe Aeromachini so far. Although sympatric species *A.* *jhora* and *A. propinquus* harbor the same *Wolbachia* strains based on MLST typing, we cannot confirm *Wolbachia* spread through introgressive hybridization based on the ML trees constructed with mt+nDNA, mtDNA, and nDNA using IQtree (Supplementary Figure S2). We also found the topological structure based on mtDNA sequence was consistent with mt+nDNA, but different from nDNA. The discordance between these patterns may have several reasons including inaccurate species taxonomy, paralogous pseudogenes, incomplete lineage sorting (ILS), and introgressive hybridization. We can exclude the possibility of inaccurate species taxonomy and paralogous pseudogenes in our case, as all specimens were identified carefully by experts and all sequences were checked for paralogous pseudogenes prior to analysis. However, we cannot really distinguish ILS from introgressive hybridization on the evidence we have so far. Also, the few substitutions detected in the nuclear markers tested here make it difficult to use these data to reconstruct fine-scale phylogenies. However, since most butterfly larvae feed on plant tissue, and adults obtain nectar from flowers or tree sap, the close relationship between butterflies and host plants might lead to infection transmission through plant mediation (Sintupachee *et al.* 2006). There are many known hymenopteran parasitoids found on both lepidopteran and dipteran hosts, and generalist parasitoids may also have mediated horizontal transmission (Apiwathnasorn 2012). This could be further tested by examining *Wolbachia* strains in parasitoids particularly in those from Yunnan province.

The recombination analysis of each MLST allele and *wsp* using RDP3 found intragenic recombination in the *FtsZ* gene in four *Wolbachia* strains. This result also argues for horizontal transmission between *Wolbachia* strains in the tribe Aeromachini; the very similar recombined *FtsZ* sequence in four species-specific *Wolbachia* strains may reflect a second horizontal transmission in these closely related species (Supplementary Figure S1). In our reconstructed ML trees for each MLST allele and *wsp* gene (Figure 4), we found potential recombination events by checking every allele for supergroup localization among the gene trees. Eleven *Wolbachia* strains from Aeromachini species showed inconsistent supergroup localization for the five MLST allele trees. The substantial incongruence between the *Wolbachia* phylogenies based on the MLST concatenated sequences and the *wsp* gene (Figure 4) suggests that the different *Wolbachia* genes have undergone independent evolutionary trajectories. This has also been observed in rice planthoppers, butterflies, and moths (Zhang *et al.* 2013; Ilinsky and Kosterin 2017) and highlights the limitations of the MLST system for classifying *Wolbachia* strains, whereas full genome sequencing may be required to further establish relationships among *Wolbachia* strains (Conner *et al.* 2017; Cooper *et al.* 2019; Meany *et al.* 2019).

Taken together, this study provides a conservative estimate of *Wolbachia* prevalence (25%) of the butterfly tribe Aeromachini with a species incidence of >46%. The cophylogenetic comparison, divergence time estimation, and *Wolbachia* recombination analysis revealed that *Wolbachia* acquisition in Aeromachini is often through horizontal transmission as also found for other groups such as fruit flies (Turelli *et al.* 2018), spiders (Baldo *et al.* 2008), wasps (Huigens *et al.* 2004), trypetids (Schuler *et al.* 2013), leaf beetles (Jackel *et al.* 2013), moths (Ahmed *et al.* 2016), rice planthoppers (Zhang *et al.* 2013), and mosquitoes (Shaikevich *et al.* 2019).

## Supplementary Material

jkab221_Supplementary_Data
