# Supplementary material for: Horizontal transmission and recombination of Wolbachia in the butterfly tribe Aeromachini Tutt, 1906 (Lepidoptera: Hesperiidae)
Source: G3 (Bethesda). 2021 Jul 1;11(9):jkab221. doi: 10.1093/g3journal/jkab221 (PMC8496314; doi:10.1093/g3journal/jkab221)
Supplement: jkab221_Supplementary_Data [file jkab221_supplementary_data.zip › jkab221-suppl_data/GENETICS-G3-2021-402635-s06.docx]

Figure S1. Description of recombination events. For each alignment, only polymorphic sites around the breakpoints are shown. Numbers above sequence alignments indicate the schematic nucleotide position. Percentages above and under the sequence alignments show the similarities of the daughter sequence to its major and minor parent sequences (marked with the same background color).

Figure S2. Maximum likelihood trees for mtDNA + nDNA, mtDNA and nDNA. Numbers beside nodes are IQTREE ultrafast bootstrap and SH-aLRT values.
