# Supplementary figures and images for: Horizontal transmission and recombination of Wolbachia in the butterfly tribe Aeromachini Tutt, 1906 (Lepidoptera: Hesperiidae)
Source: G3 (Bethesda). 2021 Jul 1;11(9):jkab221. doi: 10.1093/g3journal/jkab221 (PMC8496314; doi:10.1093/g3journal/jkab221)

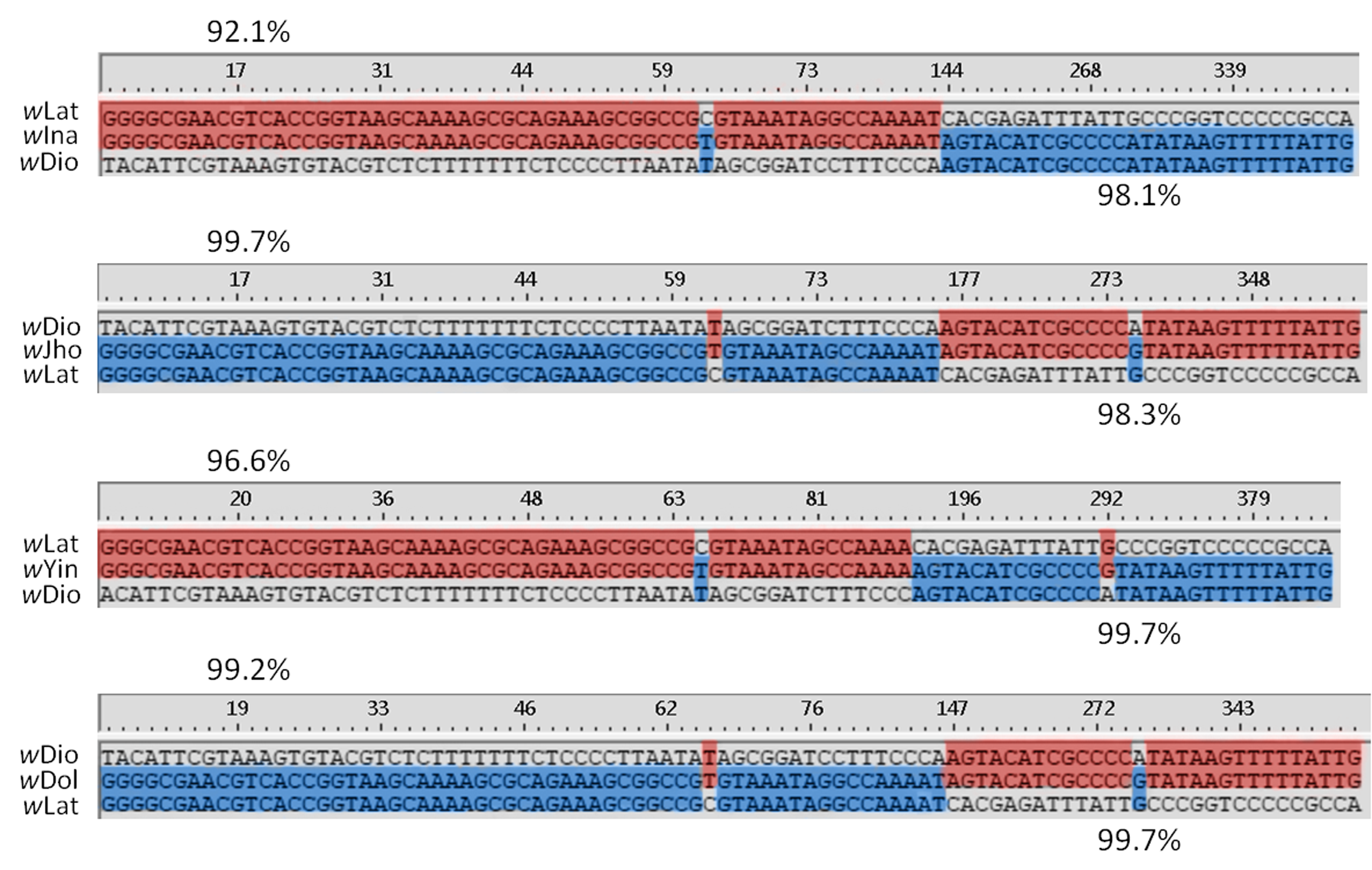

Supplement: jkab221_Supplementary_Data [file jkab221_supplementary_data.zip › jkab221-suppl_data/GENETICS-G3-2021-402635-s01.tif]

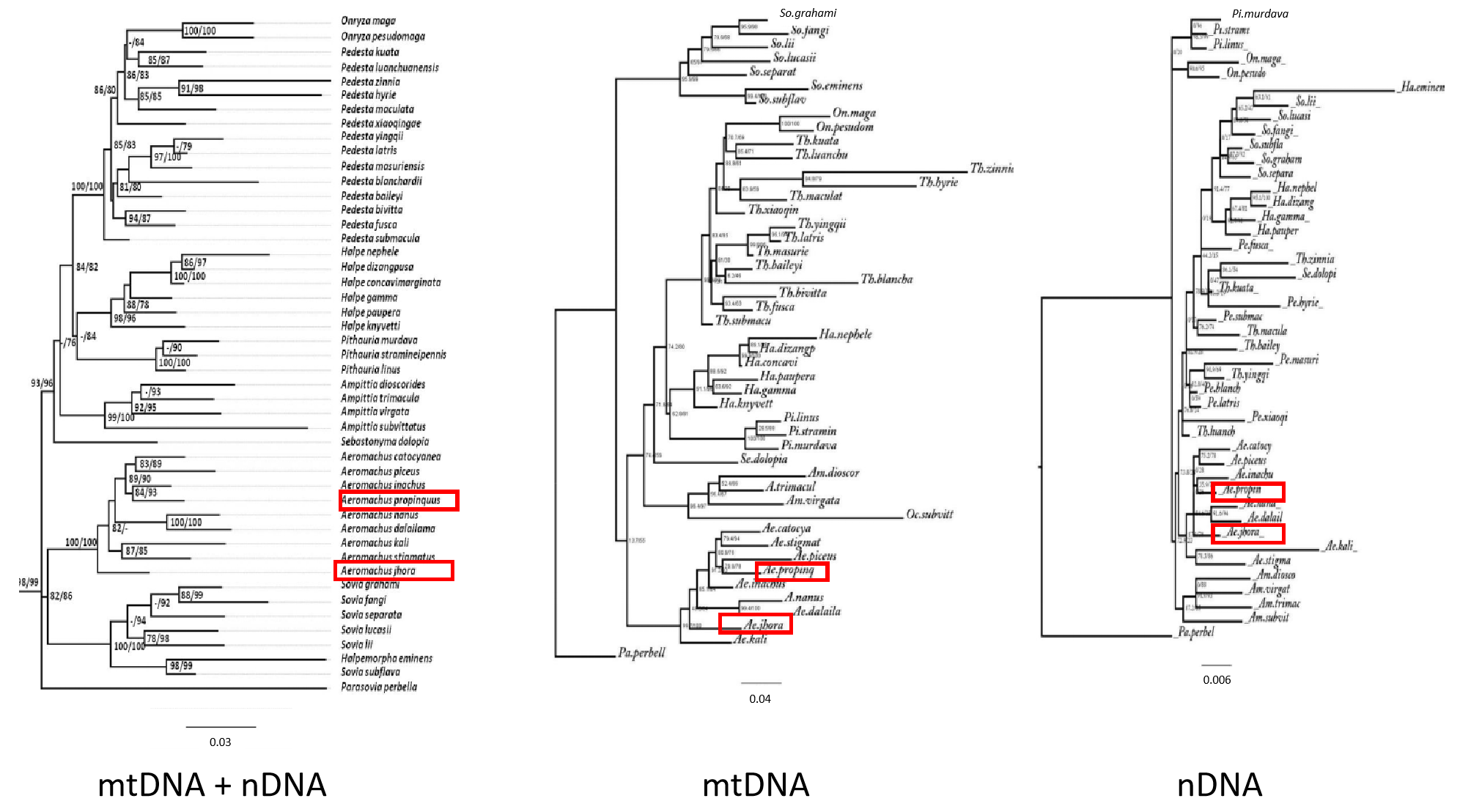

Supplement: jkab221_Supplementary_Data [file jkab221_supplementary_data.zip › jkab221-suppl_data/GENETICS-G3-2021-402635-s02.tif]
