# Supplementary material for: Horizontal transmission and recombination of Wolbachia in the butterfly tribe Aeromachini Tutt, 1906 (Lepidoptera: Hesperiidae)
Source: G3 (Bethesda). 2021 Jul 1;11(9):jkab221. doi: 10.1093/g3journal/jkab221 (PMC8496314; doi:10.1093/g3journal/jkab221)
Supplement: jkab221_Supplementary_Data [file jkab221_supplementary_data.zip › jkab221-suppl_data/GENETICS-G3-2021-402635-s04.doc]

Table S2. Primer sequences and amplicon lengths of PCR products of target genes

| Gene/region | Primers | Sequence (5’– 3’) | Amplicon length | Annealing temperature | Reference |
| --- | --- | --- | --- | --- | --- |
| *wsp* | 81_F  691_R | TGG TCC AAT AAG TGA TGA AGA AAC  AAA AAT TAA ACG CTA CTC CA | 590 ~ 630 bp | 55°C | Zhou et al., 1998 |
| *gatB* | gatB_F1  gatB_R1 | GAK TTA AAY CGY GCA GGB GTT  TGG YAA YTC RGG YAA AGA TGA | 471 bp | 54°C | Baldo et al., 2006 |
| *coxA* | coxA_F1  coxA_R1 | TTG GRG CRA TYA ACT TTA TAG  TCT AAA GAC TTT KAC RCC AGT | 487 bp | 54°C | Baldo et al., 2006 |
| *hcpA* | coxA_F1  coxA_R1 | GAA ATA RCA GTT GCT GCA AA  GAA AGT YRA GCA AGY TCT G | 515 bp | 54°C | Baldo et al., 2006 |
| *ftsZ* | ftsZ_F1  ftsZ_R1 | ATY ATG GAR CAT ATA AAR GAT AG TCR AGY AAT GGA TTR GAT AT | 524 bp | 54°C | Baldo et al., 2006 |
| *fbpA* | fbpA_F1  fbpA_R1 | GCT GCT CCR CTT GGY WTG AT  CCR CCA GAR AAA AYY ACT ATT C | 509 bp | 59°C | Baldo et al., 2006 |
